# Survey of User Needs: Mobile Apps for mHealth and People with Disabilities

**DOI:** 10.1007/978-3-030-58805-2_32

**Published:** 2020-08-12

**Authors:** Ben Lippincot, Nicole Thompson, John Morris, Mike Jones, Frank DeRuyter

**Affiliations:** 8 Institute Integriert Studieren, JKU Linz, Linz, Austria; 9 Jack Baskin School of Engineering, UC Santa Cruz, Santa Cruz, CA USA; 10 Dipartimento di Meccanica, Politecnico di Milano, Milan, Italy; 11 Support Centre for Students with Special Needs, Masaryk University Brno, Brno, Czech Republic; 12 Crawford Research Institute, Shepherd Center, Atlanta, GA 30309 USA; 13 Duke University Medical Center, Durham, NC 27705 USA

**Keywords:** mHealth, Mobile apps, User needs, Survey research

## Abstract

This paper presents data and analysis from survey research conducted by the Rehabilitation Engineering Research Center on Information and Communication Technology Access for Mobile Rehabilitation (mRehab RERC) on the use and unmet needs for mHealth mobile apps by people with disabilities in the United States. Quantitative and qualitative data are reported on user experiences with mHealth apps to map the behavior, interests and needs of people with specific types of disability (physical, cognitive, sensory, emotional/psychological, and speech). Summary results are presented for all respondents and each disability type. Slightly more than half of the participants in this sample (53.2%) reported using mHealth apps. Fitness and exercise apps were the mHealth apps most used by respondents with disabilities, followed by hospital/clinical portal apps. Symptom and disease management apps are the least commonly used, even though these would seem to be important for people with chronic conditions. Text-based responses regarding unmet needs for mHealth apps can be sorted into accessibility needs and functionality needs. In general, respondents with sensory limitations were more likely to identify accessibility needs. However, all disability groups identified both types of unmet needs. These results can help inform research and development efforts to provide mHealth apps that meet the needs of people with disabilities.

## Introduction

This article presents data and analysis from survey research conducted by the Rehabilitation Engineering Research Center on Information and Communication Technology Access for Mobile Rehabilitation (mRehab RERC) on the use and unmet needs for mHealth mobile apps by people with disabilities in the United States. The paper focuses on several key questions related to the user experience with mobile health apps in order to map the behavior, interests and needs of people with specific types of disability (physical, cognitive, sensory, emotional/psychological, and speech). Survey response data for the following questions are presented and discussed:

- Do you use any mHealth apps?
- Which types of mHealth apps do you use to maintain your health? (exercise and fitness, diet and nutrition, lifestyle and stress, clinical portals, and disease/symptom management)
- Which specific mHealth apps do you use?
- What do you want in an mHealth app that you currently have not found to meet your needs? (open-ended question)

Summary results for all respondents are presented and for each disability. This analysis provides a detailed view of the use and unmet needs for mHealth mobile apps by people with disabilities in the United States and possibly by extension in other countries.

## Background

Consumers and healthcare providers have considerable interest and high expectations for mHealth [1]. About half of patients recently surveyed in the United States predict that mHealth technologies will improve the convenience, cost and quality of healthcare in the next three years [2], and 96% of current mHealth app users believe the apps help improve their quality of life [3]. Six in 10 doctors and payers believe that their widespread adoption is inevitable, and 7 in 10 believe health apps will encourage patients to take more responsibility for their health [4].

The opportunities offered by mHealth technologies are substantial. The World Health Organization (WHO) views “digital health” solutions as a key tool to strengthen national health systems to support the goal of Universal Health Coverage (UHC). According to the WHO: “Digital technologies provide concrete opportunities to tackle health system challenges, and thereby offer the potential to enhance the coverage and quality of health practices and services” [5].

Despite the expected benefits of mHealth early evidence suggests that people with disabilities are not well represented in the growth of mobile healthcare, and particularly the proliferation of mobile health software applications (mHealth apps) for smartphones and tablets [6, 7]. This underrepresentation could widen health disparities between the general population and people with disabilities, and perhaps more fundamentally fail to take advantage of new and effective ways of engagement in personal health management.

## Methodology

The mRehab RERC staff at Shepherd Center in Atlanta, Georgia USA has conducted user needs research with people with disabilities on assistive and accessible technology since 2001. We pioneered the “network model” of user-centered research with people with disabilities with the creation in that year of our Consumer Advisory Network (CAN), a national network of people with all types of disabilities and diverse demographic backgrounds [8–10]. This network model involves two levels of user-centered research: 1) national survey research involving the entire network, and 2) small-n narrowly focused research on specific questions related to assistive and accessible technology.

Since 2001 RERC research staff has conducted national survey research on smart phone use by people with disabilities, hearing aid compatibility (HAC) of mobile phones, use of mobile phones by elders, wearable technology, smart speakers and smart home technology, among other lines of inquiry. In 2017 our staff replicated this model to facilitate user-centered research with people with disabilities for Microsoft by establishing the established the Accessibility User Research Collective (AURC) [10].

More recently our team has developed and implemented a research agenda related to mHealth and related mobile apps and technologies. Building on this base of knowledge, the study team drafted a new survey in January 2020 focusing on the mHealth apps (general types and specific apps) used by people with disabilities and, as importantly, how people with disabilities find useful and usable mHealth apps. The study team solicited input on questionnaire design from our external advisors with disabilities and other professionals who work with people with disabilities and our mRehab RERC colleagues. The questionnaire consists of 45 questions organized in the following sections:

1. Demographics
2. Disability and use of assistive technology
3. Use of mobile devices and apps
4. Use of mHealth mobile apps
5. Discovering and using new mobile apps

Data were collected from April 14 to June 10, 2020. Participants were recruited primarily through the Consumer Advisory Network (CAN) developed and maintained since 2001. We also recruited via other disability organizations in the United States with which we have collaborated for many years and through the researchers’ personal networks of people with disabilities. Data were collected in January and February 2019 using convenience sampling methods and online data collection on the Survey Monkey web-based platform. Although no protected health information (PHI) was collected in this survey, the Survey Monkey platform does meet the privacy and security requirements of the United States Health Insurance Portability and Accountability Act of 1996 (HIPAA), which establishes essential policies and practices for protecting patient health information from unnecessary and unauthorized access.

## Results

Response data were analyzed using SPSS version 22. A total of 412 individuals with various types of disability, including physical, sensory, cognitive, emotional and speech limitations, responded to our requests for participation.

Mean age of respondents in our sample is 51.1 years with a standard deviation of 15.5 years, indicating that approximately two-thirds of the sample is between the ages of 35 and 67 (Table 1). Approximately 79% of respondents identified as white/Anglo, which is somewhat higher than the national average for the general population in the United States. Just over half the sample is female, reflecting very closely the gender distribution for the general population. Slightly more than 2 in 5 respondents (41%) reported annual household income of $50,000 or higher, which is below the national median household income for the general population and seems appropriate for the population of people with disabilities due to more limited employment opportunities.

**Table 1.** Demographic background of respondents (n = 412).

Age – mean (years)	51.1
Age - standard deviation (years)	15.5
Race/ethnicity (% white/Anglo)	79.4
Gender (% female)	51.7
Education (% completed bachelor’s degree or higher)	65.8
Annual household income (% $50,000 or higher)	41.0

Table 2 shows the functional limitations included in the survey questionnaire. Notably, many respondents reported having more than one functional limitation, which is due to the likelihood that people will have multiple comorbidities resulting from a single injury, disease or chronic condition. For instance, difficulty walking is correlated with difficulty using arms and/or hands.

**Table 2.** Functional difficulties of respondents.

Disability type	Number	Percent
Worry, nervousness, anxiety	50	12.1
Difficulty thinking	50	12.1
Difficulty speaking	19	4.6
Difficulty learning	34	8.3
Difficulty using arms	44	10.7
Difficulty using hands, fingers	62	15.0
Difficulty walking, standing	98	23.8
Fatigue or limited stamina	59	14.3
Low vision (even with glasses)	60	14.6
Blind (without usable vision)	112	27.2
Hard of hearing	84	20.4
Deaf (unable to hear)	50	12.1

Slightly more than half of the participants in this sample (53.2%) report using mHealth apps. Two categories of mHealth apps – fitness/exercise and hospital/clinical portal apps – are the most used by respondents, followed by lifestyle, diet/nutrition, and disease management apps (Table 3).

**Table 3.** Types of mHealth apps used by respondents (percentage of respondents).

Disability type	Fitness, exercise	Diet, nutrition	Lifestyle, stress, sleep	Hospital or clinic portal	Disease, symptom mgmt.
Worry, nervousness, anxiety	42.0	20.0	36.0	32.0	10.0
Difficulty thinking	40.0	24.0	30.0	36.0	12.0
Difficulty speaking	15.8	15.8	10.5	26.3	15.8
Difficulty learning	35.3	26.5	26.5	38.2	8.8
Difficulty using arms	20.5	15.9	15.9	34.1	11.4
Difficulty using hands, fingers	25.8	14.5	17.5	30.6	17.7
Difficulty walking, standing	26.5	13.3	15.3	28.6	16.3
Fatigue or limited stamina	35.6	28.0	23.7	33.9	22.0
Low vision (even with glasses)	41.7	23.3	20.0	30.0	46.7
Blind (without usable vision)	46.4	18.8	20.5	30.4	11.6
Hard of hearing	38.1	11.9	15.5	26.2	9.5
Deaf (unable to hear)	42.0	12.0	20.0	26.0	6.0

These results suggest that people with disabilities use many of the same types of apps used by the general population, likely reflecting the commercial availability of these apps. Fitness and exercise apps are numerous and are built into major smartphones running the iOS and Android operating systems. Also, in the United States many hospital systems offer and promote the use of clinical portal apps that allow for scheduling medical visits, accessing laboratory results, tracking vital signs, and communicating asynchronously with healthcare providers. Indeed, the most frequently used app was MyChart, the patient portal owned by and integrated into Epic, electronic medical record platform most widely used by hospitals and health systems in the United States. MyChart claims to have over 100 million users. Among fitness apps, Fitbit and Under Armour’s MyFitnessPal were the most commonly used.

Notably, disease and symptom management apps are the least commonly used type of apps reported by respondents. One might expect that such apps would attract considerable interest among people with disabilities. These results might suggest that such apps do not enjoy the technical and marketing support of general fitness apps and patient portal apps.

Specific observations can be made about app use by people with specific disabilities. Individuals with blindness are most likely to use mHealth apps to track their fitness and exercise. Those who experience chronic fatigue and limited stamina use diet and nutrition apps most. Individuals who reported frequent worrying, nervousness and anxiety report use mHealth apps for lifestyle, stress, and sleep the most. Those with low vision, use disease/symptom management apps the most by a wide margin.

Table 4 summarizes responses to the open-ended question on the types of features, functions, and entire apps that respondents would like to have, but so far have not found in the app marketplaces. These responses can be sorted into accessibility needs (e.g., picture-based calorie counter, haptic feedback) and needs for specific functionality (e.g., sync health information from multiple healthcare providers, diabetes monitor that allows users to enter notes). In general, respondents with sensory limitations focused more on accessibility than did respondents with other disabilities. Still, respondents in each of the disability groups identified needs for both enhanced access and expanded functionality.

**Table 4.** Needs for mHealth apps identified by disability type.

Disability type	Needs for mHealth apps
Worry, nervousness, anxiety	• Guaranteed privacy • Goal and habit tracking • Universal app - all health/medical in one place
Difficulty thinking	• Picture-based calorie counter • Widgets on home screen to track health symptoms • Simple medication management system • Sync health information from multiple providers
Difficulty speaking	• Reputable review of apps by doctor or organization
Difficulty learning	• Ability to build personalized fitness regimen • Real-time suggestions based on health data/progress
Difficulty using arms	• Ability to measure heart rate and temperature • Ability monitor pain management, seizures, and O2 • Better movement monitoring for wheelchair users
Difficulty using hands, fingers	• App to schedule appointments • Apps to measure and track blood measure • Alerts when health measures out of normal range
Difficulty walking, standing	• Accuracy measuring physical activity/movement • App about spinal cord injury • Suggestions for wheelchair users
Fatigue or limited stamina	• Better nutrition app • Diabetes monitor that allows entering notes • Homeopathic, naturopathic, organic medicines info
Low vision (even with glasses)	• Accessible health records and exportable data • Gamification of exercises, like walking
Blind (without usable vision)	• Accessibility (labeled buttons and links) • Exportable personal health data • Less cluttered way to access step count • Exercise app that describes the exercises
Hard of hearing	• Haptic feedback controls • Real-time speech to text conversion
Deaf (unable to hear)	• Virtual clinic visits with captioning, ASL interpreter

## Conclusion

Mobile health apps are an essential element in the mHealth technology ecosystem. Their variety and ubiquity endow them with considerable potential to improve health and expand healthcare access. Yet, most are designed and engineered by and for people without disabilities – people without substantial accessibility challenges and with neurotypical body functioning (normal blood pressure, heart rate, calorie consumption, gait, etc.). Concerted and continuous efforts to identify the experiences and needs for mobile health apps by people disabilities is critical to realizing the potential for expanded inclusion offered by mHealth/eHealth.

This survey of consumers with disabilities and mHealth mobile apps serves as the cornerstone for our ongoing effort to track consumer use patterns and preferences, and it supports our efforts to provide information on the usability of mHealth apps by people with specific disabilities directly to these same consumers. In order to respond to the rapid pace of change in consumer technology and track consumer perceptions, experiences and preferences over time, we plan to update, refine and conduct this key consumer survey regularly.

Survey results also support two key initiatives of the mRehab RERC: 1) provide an mHealth app accessibility clearinghouse with consumer reviews of specific mHealth apps; and 2) conduct an annual call for proposals from external developers working in the area of mHealth apps for people with disabilities. We will use the information gathered through the mHealth apps survey to identify promising apps worthy of in-depth testing and to help inform the types of mHealth apps we are most interested in funding for development each year.
